# Modulation of host central carbon metabolism and *in situ* glucose uptake by intracellular *Trypanosoma cruzi* amastigotes

**DOI:** 10.1371/journal.ppat.1006747

**Published:** 2017-11-27

**Authors:** Sheena Shah-Simpson, Gaelle Lentini, Peter C. Dumoulin, Barbara A. Burleigh

**Affiliations:** Department of Immunology and Infectious Diseases, Harvard T. H. Chan School of Public Health, Boston, Massachusetts, United States of America; Oregon Health & Science University, UNITED STATES

## Abstract

Obligate intracellular pathogens satisfy their nutrient requirements by coupling to host metabolic processes, often modulating these pathways to facilitate access to key metabolites. Such metabolic dependencies represent potential targets for pathogen control, but remain largely uncharacterized for the intracellular protozoan parasite and causative agent of Chagas disease, *Trypanosoma cruzi*. Perturbations in host central carbon and energy metabolism have been reported in mammalian *T*. *cruzi* infection, with no information regarding the impact of host metabolic changes on the intracellular amastigote life stage. Here, we performed cell-based studies to elucidate the interplay between infection with intracellular *T*. *cruzi* amastigotes and host cellular energy metabolism. *T*. *cruzi* infection of non-phagocytic cells was characterized by increased glucose uptake into infected cells and increased mitochondrial respiration and mitochondrial biogenesis. While intracellular amastigote growth was unaffected by decreased host respiratory capacity, restriction of extracellular glucose impaired amastigote proliferation and sensitized parasites to further growth inhibition by 2-deoxyglucose. These observations led us to consider whether intracellular *T*. *cruzi* amastigotes utilize glucose directly as a substrate to fuel metabolism. Consistent with this prediction, isolated *T*. *cruzi* amastigotes transport extracellular glucose with kinetics similar to trypomastigotes, with subsequent metabolism as demonstrated in ^13^C-glucose labeling and substrate utilization assays. Metabolic labeling of *T*. *cruzi*-infected cells further demonstrated the ability of intracellular parasites to access host hexose pools *in situ*. These findings are consistent with a model in which intracellular *T*. *cruzi* amastigotes capitalize on the host metabolic response to parasite infection, including the increase in glucose uptake, to fuel their own metabolism and replication in the host cytosol. Our findings enrich current views regarding available carbon sources for intracellular *T*. *cruzi* amastigotes and underscore the metabolic flexibility of this pathogen, a feature predicted to underlie successful colonization of tissues with distinct metabolic profiles in the mammalian host.

## Author summary

The kinetoplastid protozoan, *Trypanosoma cruzi*, is a highly successful parasite with a broad mammalian host range and the capacity to colonize a variety of tissues within a given host to establish life-long infection. *T*. *cruzi* infection causes Chagas disease in humans, characterized by severe cardiomyopathy and gastrointestinal motility disorders, with limited treatment options. Despite the critical role of *T*. *cruzi* amastigotes in sustaining mammalian infection, little is known about their metabolic requirements or the range of nutrients available to these parasites in the host cell cytoplasm. Here, we demonstrate that *T*. *cruzi* infection triggers a host response in infected cells that includes increased mitochondrial respiration and biogenesis and increased glucose uptake into infected cells. We show that exogenous glucose supports optimal intracellular parasite replication and that cytosolic *T*. *cruzi* amastigotes access glucose *in situ*, potentially via a facilitated transport process characterized here. These findings expand our view of the range of carbons available to the intracellular parasite and suggest even greater metabolic flexibility of the tissue-infective stages of *T*. *cruzi* than previously appreciated, a capability predicted to contribute to successful host colonization.

## Introduction

Chagas disease is a vector-borne parasitic disease caused by the kinetoplastid protozoan parasite *Trypanosoma cruzi*. Acute *T*. *cruzi* infection is most often asymptomatic or characterized by flu-like symptoms, but can cause severe and fatal myocarditis in the first weeks following infection [1]. More typically, parasites establish chronic infection that is controlled, but not eliminated, by host immune mechanisms [2]. A subset of chronically infected individuals develop progressive disease characterized by serious cardiac and gastrointestinal disturbances [3], for which treatment options are limited [4]. *T*. *cruzi* exhibits a broad mammalian host range where it can colonize diverse tissue types [5, 6]. In the chronic stage of infection, when parasites are maintained at very low densities, persistence has been reported most often in cardiac muscle, gastrointestinal smooth muscle and adipose tissue [6–9]. Knowledge of the molecular mechanisms governing successful intracellular colonization, replication and long-term persistence by *T*. *cruzi* are currently lacking but represent potentially exploitable processes for the development of new therapeutics.

In mammalian hosts, *T*. *cruzi* transitions between two main developmental forms. The non-dividing, motile trypomastigote can actively penetrate most nucleated cell types by exploiting the host cell plasma membrane repair process [10]. Once inside the host cell, the parasite sheds its temporary vacuole [11] and progresses through a developmental program that culminates in the formation of the morphologically and biochemically distinct amastigote form that replicates in the host cell cytosol. Transcriptomic profiling of this developmental transition revealed strong signatures of global metabolic reprogramming in the parasite as it transforms from the trypomastigote to the amastigote stage [12]. As an obligate intracellular parasite, *T*. *cruzi* amastigotes are forced to draw from host nutrient pools to fuel their growth and survival, although nutrient uptake by amastigotes *in situ* has not been directly demonstrated. On the basis of expression data [12–14], functional studies [15], and metabolic assays conducted with isolated amastigotes [16, 17], it has been proposed that amino acids and fatty acids are the most likely sources of carbon for *T*. *cruzi* amastigotes to fuel their metabolism. Hexose sugars have largely been discounted as a potential carbon source for this cytosolic pathogen [13, 15, 18], due to the perception that glucose is a negligible commodity in the interior of a mammalian host cell [18] and the failure to demonstrate hexose transporter expression or uptake in isolated *T*. *cruzi* amastigotes [13, 15]. Nevertheless, the ability of *T*. *cruzi* to colonize a wide range of cell and tissue types predicts a degree of metabolic flexibility and/or the potential for the parasite to reprogram host metabolic pathways to suit its specific metabolic requirements as reported with some viral and bacterial pathogens [19–21].

Metabolic abnormalities have been reported in chronic Chagas patients [22–24] and in animal models of acute and chronic *T*. *cruzi* infection [25–29]. These include dysregulation of glucose [23, 28] and lipid metabolism [29] as well as mitochondrial electron transport chain dysfunction [26, 27, 30] in *T*. *cruzi*-infected skeletal [31] and cardiac muscle [25]. Recently, metabolite profiling studies have revealed increased uptake and metabolism of glucose in *T*. *cruzi-*infected cardiac muscle [32]. At the cellular level, transcriptomic analyses reveal modulation of host metabolic pathway expression including upregulation of metabolite transporters in *T*. *cruzi-*infected fibroblasts [12, 33]. How such metabolic changes in the host impact the intracellular *T*. *cruzi* amastigote life cycle have not been determined. However, results of genome-scale functional studies predict that the immediate metabolic environment can influence intracellular parasite growth [34]. In the present study, we sought to determine how *T*. *cruzi* infection impacts host glucose metabolism and mitochondrial respiration at the cellular level and how parasite-triggered changes in host cellular metabolism influence the intracellular infection cycle.

## Results

### Glucose uptake and mitochondrial respiration are elevated in *T*. *cruzi*-infected host cells

**Fig 1:**
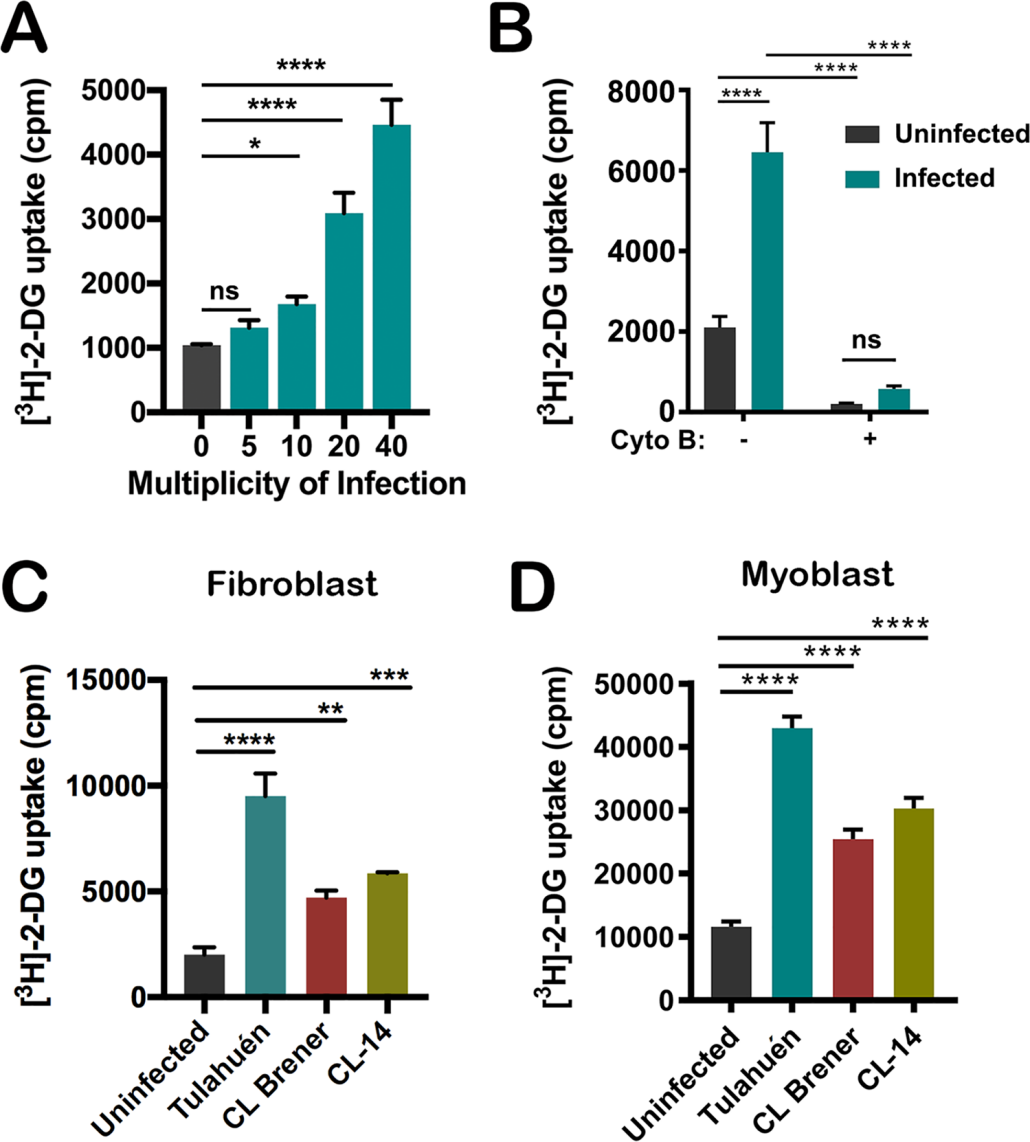
*T*. *cruzi* infection increases glucose uptake by host cells. **(A)** Uptake of [^3^H]-2-deoxyglucose ([^3^H]-2-DG) into uninfected or *T*. *cruzi*-infected NHDF monolayers at 48 hours post infection (48 hpi), in which infection was established with a varying multiplicity of infection (MOI). Mean ± SD shown for 3 biological replicates per MOI. One-way ANOVA with Dunnett’s multiple comparison test was applied for individual comparisons to the uninfected control group (*p<0.05, ****p<0.0001). **(B)** Cytochalasin B (10 μM) blocks uptake of [^3^H]-2-DG in uninfected and infected NHDF monolayers (48 hpi). Mean ± SD shown for 3 biological replicates. Two-way ANOVA with Tukey’s multiple comparisons test was applied (****p<. 0001)**. (C)** [^3^H]-2-DG uptake by NHDF or **(D)** C2C12 myoblasts following a 48 h infection with *T*. *cruzi* Tulahuén, CL Brener or CL-14 strains. NHDF were infected for 2 hours with MOI 40 for Tulahuén strain and MOI 150 for CL Brener and CL-14 strains. C2C12 were infected for 2 hours with MOI 80 for Tulahuén strain and MOI 150 for CL Brener and CL-14 strains. Mean ± SD for 3 biological replicates. One-way ANOVA with Dunnett’s multiple comparison test was applied for individual comparisons to the uninfected control group (**p< 0.01, ***p< 0.001, ****p< 0.0001).

Metabolite profiling of *T*. *cruzi*-infected hearts has provided evidence of increased glucose uptake and metabolism at the whole organ level [32]. Here, we examined the impact of *T*. *cruzi* infection on glucose metabolism at the cellular level using low passage normal human dermal fibroblasts (NHDF), which have been shown to increase host glucose transporter expression during *T*. *cruzi* infection [12, 33]. Following infection (48 hpi) we observed a significant increase in 2-deoxyglucose ([^3^H]-2-DG) uptake into infected fibroblast monolayers in a manner that correlated with increasing parasite load (Fig 1A) under conditions where host cell abundance remained unchanged (S1A Fig). [^3^H]-2-DG uptake by both uninfected and infected NHDF was blocked by cytochalasin B (Fig 1B), consistent with a role for host plasma membrane glucose transporters [35] in mediating this host cell response to *T*. *cruzi* infection. Glucose uptake assays performed in parallel with fibroblasts (Fig 1C) and mouse skeletal myoblasts (Fig 1D) following infection with one of three *T*. *cruzi* strains: Tulahuén, CL Brener and CL-14, revealed comparable results with increased glucose uptake occurring in parasite-infected host cells as a generalized response among the isolates and mammalian cells tested here.

**Fig 2:**
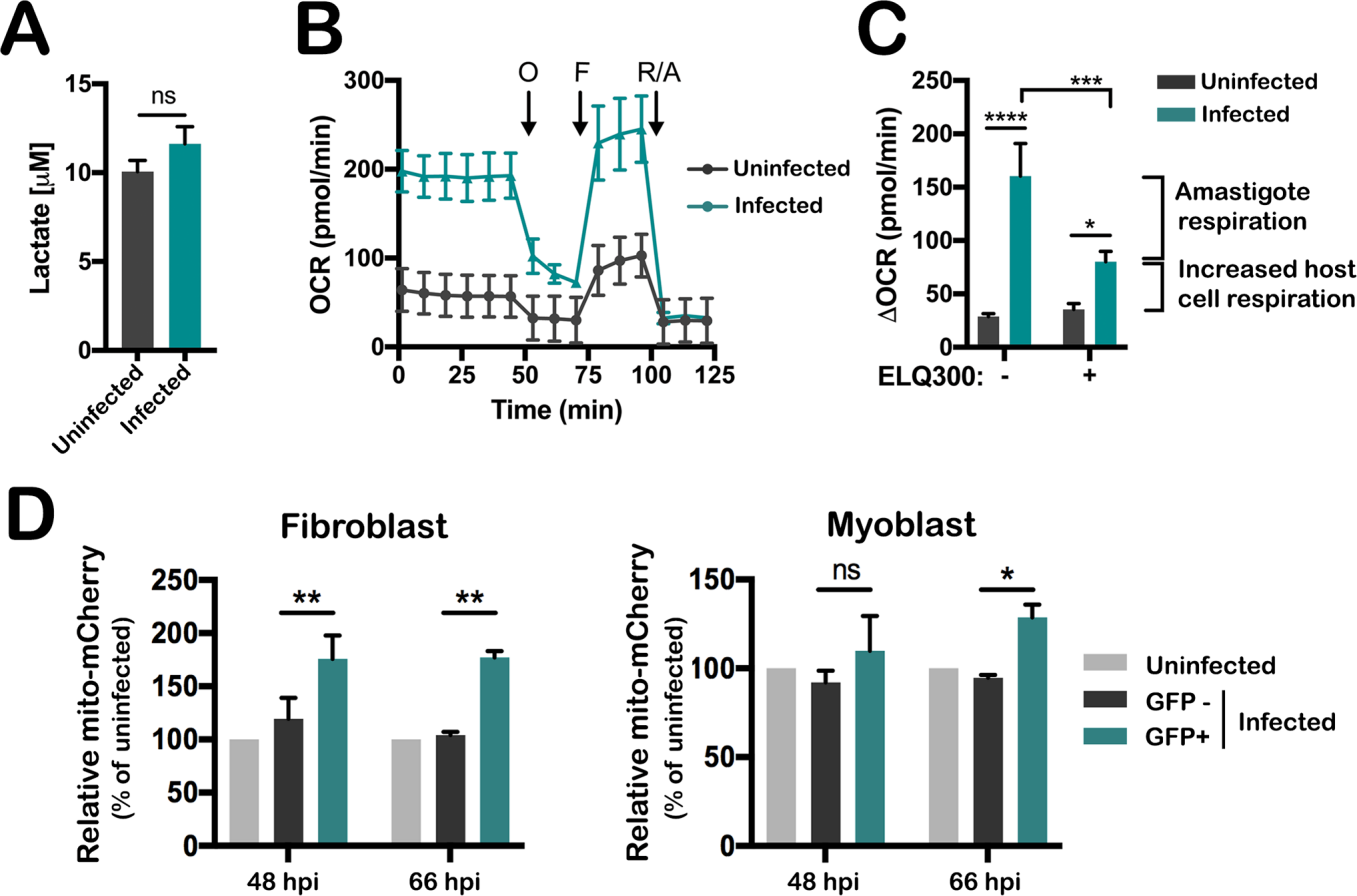
*T*. *cruzi* infection increases host mitochondrial content and respiration. **(A)** Extracellular lactate measured in culture supernatants of uninfected and *T*. *cruzi*-infected NHDF monolayers (48 hpi). Mean ± SD shown for 2 biological replicates each. Student’s t-test was applied. **(B)** Oxygen consumption rate (OCR) in uninfected and *T*. *cruzi*-infected NHDF monolayers (48hpi) before and after injection of oligomycin (O), FCCP (F), and rotenone and antimycin A (R/A). Mean ± SD shown for 4 biological replicates. **(C)** *T*. *cruzi*-infected NHDF monolayers were treated with 1 μM ELQ300 to selectively remove amastigote respiration from the total OCR signal (Infected, *±*ELQ300). Increased host respiration during *T*. *cruzi* infection (Infected, +ELQ300). Mean ± SD shown for 4 biological replicates per condition. Two-way ANOVA with Tukey’s multiple comparisons test was applied (*p<0.05, ***p<0.001, ****p<0.0001). **(D)** Geometric mean fluorescence intensity of mitochondrial mCherry signal for each condition relative to uninfected controls in NHDF and C2C12 myoblast. Infected cells were discriminated based on *T*. *cruzi* GFP expression (S2D Fig), and the geometric mean mCherry fluorescence was determined from each subpopulation (S2E Fig). Mean ± SD for 2 independent experiments. Two-way ANOVA with Dunnett’s multiple comparisons test was applied (*p< 0.05, **p< 0.01).

Glucose transport by mammalian cells is a highly regulated process [36] that is responsive to acute changes in the environment, including glucose restriction [37], intracellular pathogen infection [19–21], and acute exposure to PAMPs or cytokines [38, 39]. Physiologic triggers leading to increased glucose uptake, including pathogen infection, frequently promote increased glycolytic rates and lactate production from pyruvate, as well as decreased flux through the TCA cycle with reduced respiratory rates [40–42]. Unlike these examples, we find no increase in lactate secretion to accompany increased glucose uptake into *T*. *cruzi*-infected host cells (Fig 2A), but evidence of increased mitochondrial respiration in parasite-infected fibroblasts (Fig 2B) as determined by monitoring the oxygen consumption rate (OCR) in cell monolayers using a Seahorse extracellular flux analyzer. While the OCR measured in *T*. *cruzi*-infected monolayers was consistently greater than that measured in uninfected cell monolayers (Fig 2B), the potential for parasite respiration to contribute to the total OCR signal complicated immediate interpretation of this result. To examine this further, we sought a method to specifically inhibit *T*. *cruzi* amastigote respiration *in situ* in order to reveal the host and parasite contributions to the total OCR signal. For this, we utilized the endochin-like quinolone ELQ300, which targets cytochrome *bc*_*1*_ in the mitochondrial electron transport chain of apicomplexan parasites without affecting mammalian respiratory complexes [43, 44]. To validate the utility of ELQ300 for our purpose, we performed preliminary experiments to demonstrate that respiration in isolated *T*. *cruzi* amastigotes was inhibited by ELQ300 in a dose-dependent manner. Treatment with 1 μM ELQ300 resulted in maximal inhibition (90%) of basal OCR in isolated amastigotes (S1B Fig) with no impact on host mitochondrial respiration (S1C Fig). To confirm that ELQ300 was effective in blocking *T*. *cruzi* amastigote respiration *in situ*, we exploited a mitochondrial complex III-deficient human fibroblast cell line (CIII mutant) [45, 46] with significantly lower respiratory rates than normal human fibroblast control lines (S1D Fig). Treatment of infected CIII mutant fibroblasts with 1 μM ELQ300 almost completely abrogated the increase in OCR due to infection (S1E Fig), consistent with the compound inhibiting >90% of amastigote respiration *in situ*. In contrast, experiments performed in parallel with respiration-competent, control human fibroblasts (S1F Fig) show that following treatment with 1 μM ELQ300 to inhibit parasite respiration, a significant residual OCR signal remained, attributable to increased host cell mitochondrial respiration associated with *T*. *cruzi* infection. Similar results were obtained with *T*. *cruzi*-infected NHDF treated with 1 μM ELQ300 (Fig 2C) or with 1 μM GNF7686 (S1G–S1I Fig), a compound for which *T*. *cruzi* cytochrome *b* is the validated target [47]. Therefore, through differential targeting of *T*. *cruzi* mitochondrial complex III using small molecule inhibitors, we were able to measure intracellular amastigote respiration *in situ* and to determine that host mitochondrial respiration increases as a result of *T*. *cruzi* infection. We further demonstrate that increases in host mitochondrial respiration are accompanied by increased host mitochondrial content specifically within the parasitized subpopulation of the infected cell monolayers (Fig 2D and 2E (GFP+); S2 Fig).

### Proliferation of intracellular *T*. *cruzi* amastigotes is sensitive to exogenous glucose but not host mitochondrial respiratory capacity

**Fig 3:**
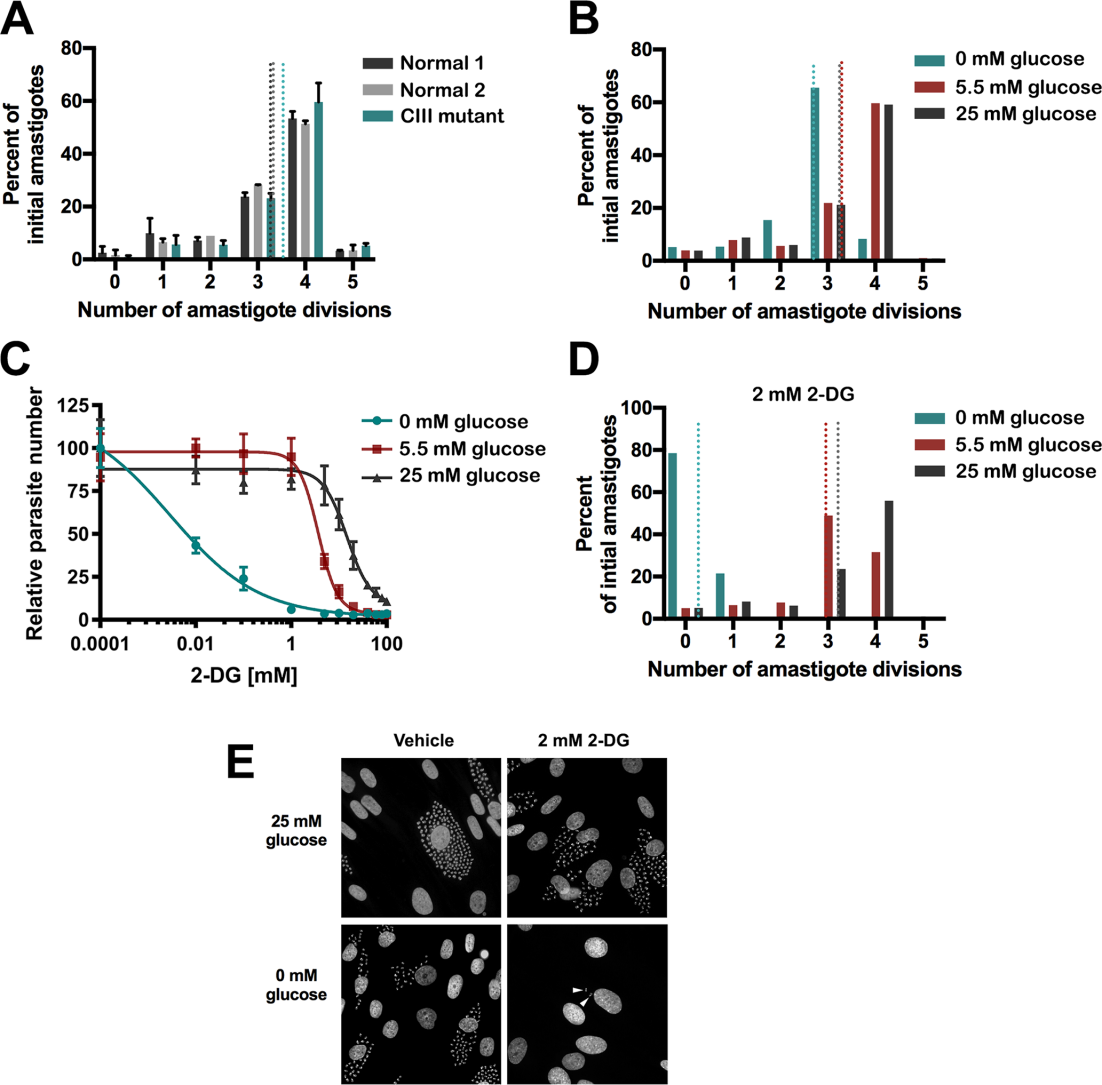
Intracellular *T*. *cruzi* replication is sensitive to exogenous glucose but not host mitochondrial electron transport chain activity. **(A)** Proliferation of *T*. *cruzi* amastigotes in human dermal fibroblasts with ETC complex III deficiency (CIII mutant) or two independent control fibroblast lines (Normal 1 and 2) derived from flow cytometric data (as detailed in Methods). Data are normalized to represent the percentage of initial amastigotes (18 hpi) that divided the indicated number of times by 48 hpi. Mean ± SD of 2 independent experiments. Dotted lines represent average number of complete amastigote divisions achieved by 48 hpi in each condition. **(B)** Proliferation of *T*. *cruzi* amastigotes in NHDF cultured in medium with varying glucose concentrations. Dotted lines represent average number of amastigote divisions achieved by 48 hpi as determined by flow cytometry of CFSE-labeled parasites. **(C)** Dose-dependent inhibition of *T*. *cruzi* growth in NHDF by 2-deoxyglucose (2-DG) in varying glucose concentrations. Relative number of *T*. *cruzi*-ß-galactosidase parasites assessed by Beta-Glo luminescence at 66 hpi shown with nonlinear fit using log(inhibitor) vs. response with variable slope. Mean ± SD of 4 biological replicates per point. **(D)** Arrest of *T*. *cruzi* amastigote proliferation in NHDF in the presence of 2 mM 2-DG under conditions of glucose depletion. Dotted lines represent average number of amastigote divisions achieved by 48 hpi as determined by flow cytometry of CFSE-labeled parasites. **(E)** Fluorescence micrographs of aldehyde-fixed, DAPI-stained NHDF monolayers corresponding to conditions used in panel D, in which host cell nuclei (large) and parasite DNA (smaller dots) are readily observed. Arrows point to 2 intracellular amastigotes that persist after severe growth restriction caused by glucose withdrawal and 2-DG treatment.

To assess the potential for altered host glucose and mitochondrial metabolism to impact intracellular amastigote replication, flow cytometry-based proliferation assays were performed that enabled determination of the number of divisions that an individual amastigote has undergone in infected host cells within a set time frame, following exposure to different conditions. Examination of *T*. *cruzi* amastigote proliferation in complex III mutant fibroblasts, which display a significantly reduced mitochondrial respiratory capacity as compared to normal fibroblasts (S1D Fig), reveals nearly identical proliferation profiles for amastigotes in CIII mutant or normal control fibroblasts (Fig 3A). In contrast, amastigote proliferation was substantially reduced when glucose was removed from the extracellular medium, with the majority of amastigotes completing only 3 divisions within 48 hours rather than 4 divisions as when glucose was present (Fig 3B). The inhibitory effect of glucose restriction on *T*. *cruzi* amastigote growth was greatly enhanced by the addition of the glucose analogue 2-deoxyglucose (2-DG) (Fig 3C), which inhibits glycolysis. Notably, the fibroblast host monolayer was not measurably impacted even in the absence of exogenous glucose, until concentrations >2 mM 2-DG were reached (S3A Fig). In the absence of exogenous glucose, amastigote proliferation was arrested by the addition of 2 mM 2-DG (Fig 3D), where the median number of parasites/infected host cell was 1 (S3B Fig). However, the continued presence of viable amastigotes in infected monolayers at 66 hpi in cultures treated with 2 mM 2-DG in the absence of glucose (Fig 3E) suggests that this effect is cytostatic rather than lethal for the parasite. The near complete arrest of intracellular *T*. *cruzi* growth in the presence of 2-DG, as opposed to the more modest effect of glucose restriction alone (Fig 3B and 3E), suggests that 2-DG may directly inhibit parasite glucose metabolism in addition to its inhibitory effect on host glycolysis. This implies that the parasite can access and internalize this glucose analog *in situ*, which counters the hypothesis that glucose is not accessible to the amastigote stage of *T*. *cruzi* [15, 18].

### Transport and catabolism of extracellular glucose by *T*. *cruzi* amastigotes

**Fig 4:**
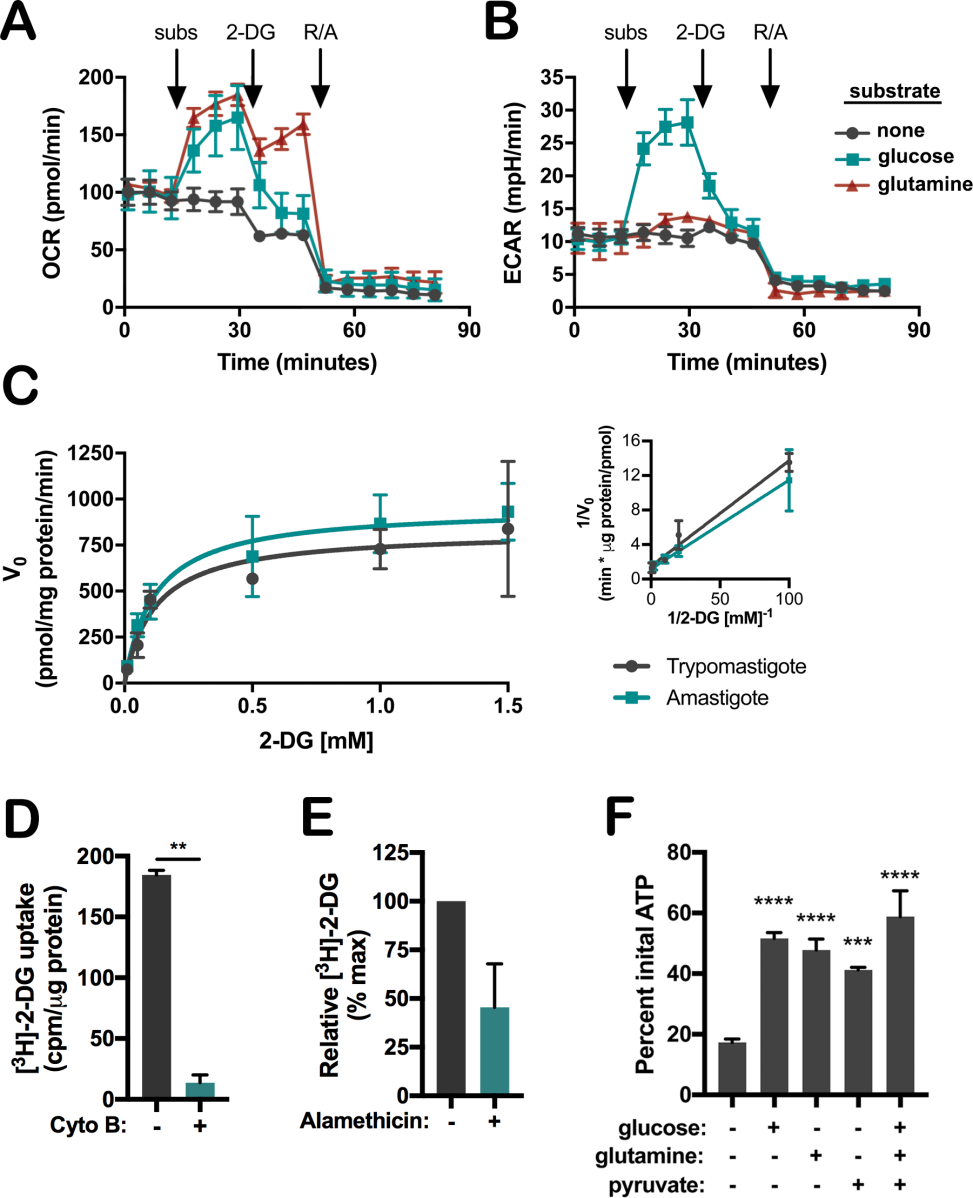
Acquisition and metabolism of glucose by intracellular *T*. *cruzi* amastigotes. Isolated *T*. *cruzi* amastigotes utilize exogenous substrates as determined by increased **(A)** oxygen consumption rate (OCR) and **(B)** extracellular acidification rate (ECAR). After establishing baseline rates, glucose (5 mM), glutamine (5 mM) or buffer were injected as substrates (subs), followed by 100 mM 2-DG to rapidly inhibit glycolysis, and 1 μM rotenone and antimycin A (R/A) to shut down mitochondrial respiration. Mean ± SD of 3 biological replicates. **(C)** Initial rate (V_0_) of [^3^H]-2-DG uptake by isolated *T*. *cruzi* amastigotes or trypomastigotes plotted for a range of substrate concentrations. Mean ± SD of two independent experiments with biological duplicates shown for each lifecycle stage. Inset shows Lineweaver-Burk plot. **(D)** Intracellular *T*. *cruzi* amastigotes access exogenous hexose *in situ*. *T*. *cruzi-*infected monolayers were incubated with 10 μCi [^3^H]-2-DG in the absence or presence of cytochalasin B (15 μM) for 20 minutes prior to isolation of intracellular amastigotes for scintillation counts. Mean ± SD of two independent experiments. Student’s t-test was applied (**p< 0.01). **(E)** [^3^H]-2-DG is internalized by intracellular amastigotes. Following isolation from monolayers pulsed with [^3^H]-2-DG, treatment of amastigotes with 0.05 mg/mL alamethicin released internalized, non-bound substrate. Mean ± SD of two independent experiments. **(F)** ATP levels measured in intracellular-derived amastigotes 24 hours after incubation with the indicated carbon substrate relative to initial ATP levels of freshly isolated parasites. Mean ± SD of 3 biological replicates per condition. One-way ANOVA with Dunnett’s multiple comparison test was applied for individual comparisons to the substrate deficient condition (***p< 0.001, ****p< 0.0001).

To explore the relationship between *T*. *cruzi* amastigotes and exogenous glucose more closely, we first examined the capacity of amastigotes, isolated from NHDF monolayers at 48 hpi, to utilize exogenous glucose to drive glycolysis and mitochondrial respiration, employing glutamine as a positive control to fuel respiration [17]. Extracellular flux analysis revealed that isolated *T*. *cruzi* amastigotes respond to exogenous glucose with significant increases in OCR (Fig 4A) and extracellular acidification rate (ECAR), which correlates with glycolytic activity, (Fig 4B) that were quenched by the injection of 2-DG. A similar increase in amastigote OCR was observed in response to glutamine, with little change in ECAR as expected (Fig 4A and 4B). To ensure that exogenous glucose is metabolized by the isolated amastigotes, rather than triggering metabolic changes in the parasite through an independent mechanism, metabolite profiling was performed following incubation of isolated amastigotes in medium containing [^13^C]-U-glucose for 3 hours. As shown in Table 1, ^13^C incorporation was detected in glycolytic and TCA cycle intermediates as well as pentose phosphate pathway intermediates and several amino acids, providing direct confirmation that *T*. *cruzi* amastigotes are capable of internalizing and metabolizing exogenous glucose in catabolic and anabolic processes.

**Table 1:** *T*. *cruzi* amastigotes incorporate exogenous glucose into multiple metabolic pathways.

Metabolite		% label incorporation ± SD
**Glycolysis**		
	glucose-6-phosphate	40.51 ± 10.29
	fructose-6-phosphate	97.49 ± 0.40
	fructose-1,6-bisphosphate	95.15 ± 0.84
	glyceraldehdye-3-phosphate	85.94 ± 1.08
	3-phosphoglycerate	92.90 ± 1.88
	phosphoenolpyruvate	93.23 ± 0.98
**TCA**		
	acetyl-CoA	78.16 ± 4.85
	citrate	83.52 ± 1.97
	α-ketoglutarate	8.89 ± 2.82
	succinate	74.95 ± 0.91
	fumarate	75.46 ± 0.77
	malate	70.79 ± 0.84
	oxaloacetate	67.36 ± 3.44
**PPP**		
	ribose-5-phosphate	63.11 ± 4.45
	sedoheptulose-7-phosphate	89.29 ± 2.66
	erythrose-4-phosphate	94.43 ± 1.55
**Amino Acids**		
	aspartate	63.88 ± 1.40
	alanine	52.48 ± 14.62
	asparagine	22.25 ± 2.54
	D-glucosamine-6-phosphate	97.72 ± 3.95
	glucosamine	30.27 ± 3.33
	glutamate	75.39 ± 2.03
	glutamine	15.17 ± 3.67
	mevalonate	79.47 ± 11.47
	N-acetyl-glutamate	33.92 ± 18.40
	N-acetyl-glutamine	49.95 ± 22.71

Isolated intracellular amastigotes were incubated with [^13^C]-U-glucose (6 mM) for 3 hours and the relative label incorporation into a specific metabolite pool was calculated. Mean ± SD of triplicates shown for a subset of metabolites representing glycolytic, tricarboxylic acid (TCA), pentose phosphate (PPP) pathways and amino acids.

Next, we performed transport assays to measure the kinetics of [^3^H]-2-DG uptake by freshly isolated intracellular amastigotes and extracellular trypomastigotes, a life cycle stage of *T*. *cruzi* for which hexose transporter expression is abundant [12, 15]. The initial rates of hexose transport (V_0_) measured for isolated amastigotes and trypomastigotes were found to be comparable, with a similar K_M_ (87.0 ± 21.7 vs. 81.2 ± 3.7 μM) and V_max_ (857.0 ± 76 vs. 666.5 ± 36.1 pmol 2-DG/mg protein/min) (Fig 4C). We then sought to determine whether the capacity for glucose uptake and metabolism by *T*. *cruzi* amastigotes is relevant in the context of an intracellular infection of mammalian cells. Infected NHDF monolayers (48 hpi) were pulsed with [^3^H]-2-DG for 20 minutes in the presence and absence of cytochalasin B, which significantly impairs glucose transport in mammalian cells but not in *T*. *cruzi* [35, 48]. Intracellular amastigotes purified from host cells showed incorporation of [^3^H] when mammalian glucose uptake was not inhibited with cytochalasin B (Fig 4D). To establish that [^3^H]-2-DG was internalized by the intracellular parasites and not non-specifically bound to amastigote surfaces following disruption of infected cells, isolated amastigotes were treated with the pore-forming peptide antibiotic, alamethicin [49] to permeabilize the parasite membrane (S4A Fig), which resulted in the release of >50% of the amastigote-associated label on average (Fig 4E). Similar evidence of *in situ* [^3^H]-2-DG uptake by intracellular *T*. *cruzi* was observed when parasites were resident in mouse skeletal myoblasts (S4B Fig). Additional examination of CL Brener (S4C Fig) and CL-14 (S4D Fig) strain amastigotes in fibroblasts provided further evidence for the internalization of [^3^H]-2-DG by intracellular *T*. *cruzi* amastigotes *in situ*. We further demonstrate that glucose, as the sole exogenous carbon source available to isolated amastigotes, is capable of sustaining ATP pools in the parasite over a 24-hour period, at levels similar to a mixture of glucose, glutamine and pyruvate (Fig 4F). Combined, these data demonstrate the potential for glucose to be utilized by intracellular *T*. *cruzi* amastigotes as a fuel for parasite metabolism *in situ*.

## Discussion

We have identified changes in host cellular metabolism associated with intracellular *T*. *cruzi* infection which include increased glucose uptake by infected host cell monolayers, increased mitochondrial respiration, and evidence of increased mitochondrial content specific to parasitized host cells. While these metabolic perturbations likely reflect multiple complex origins including compensatory changes triggered by increased metabolic demands, our data indicate that the parasite benefits from the increased glucose transport observed by infected cells, where exogenous glucose levels impact the proliferation rate of intracellular *T*. *cruzi* amastigotes. The growth-promoting effect of extracellular glucose could arise if cytosolically-localized *T*. *cruzi* amastigotes access intracellular glucose pools directly or if host cell metabolic processes fueled by extracellular glucose indirectly modulate the ability of amastigotes to efficiently replicate. While we cannot rule out the latter possibility, we provide evidence that intracellular amastigotes from different *T*. *cruzi* strains are capable of internalizing and retaining the radiolabeled glucose analog, [^3^H]-2-DG, during their intracellular replicative cycle in the mammalian cell cytoplasm. By studying amastigotes in isolation we also definitively demonstrate their capacity to take up exogenous glucose and metabolize this carbon to fuel glycolysis and mitochondrial respiration. ^13^C-glucose tracer studies confirm results of bioenergetics studies and further demonstrate that exogenous glucose is also shuttled into anabolic pathways including the pentose phosphate pathway. Combined with the finding that in the absence of other exogenous carbon sources, glucose is capable of sustaining ATP levels in isolated *T*. *cruzi* amastigotes to a similar degree as a mixture of substrates, our data indicate the potential for glucose to serve as an important substrate for the intracellular life stages of *T*. *cruzi*.

These data counter the view that glucose is unlikely to be utilized by intracellular *T*. *cruzi* parasites [18]. While glucose concentrations in the mammalian cell cytoplasm have previously been considered insufficient to support the growth of cytosolic pathogens, this argument is weakened with studies using fluorescent glucose sensors that demonstrate the existence of a significant and dynamic pool of cytosolic glucose in multiple human cell lines [50, 51]. A more direct argument against glucose as a potential substrate for intracellular *T*. *cruzi* amastigotes is the report that hexose transporter expression, as well as the ability to transport exogenous glucose, is negligible in this life cycle stage of the parasite [15]. However, our demonstration that isolated *T*. *cruzi* amastigotes transport glucose with similar kinetics as the trypomastigote stage of the parasite suggest that amastigotes utilize a facilitated hexose transporter with similar properties, if not identical, to the transporters expressed by extracellular *T*. *cruzi* life stages [52]. We further confirmed hexose transporter expression in amastigotes from three different *T*. *cruzi* strains by quantitative RT-PCR, albeit at much lower levels than in trypomastigotes. Notably, CL-14 amastigotes had the lowest expression (S5 Fig), but the capacity to take up glucose from the host cytosol was exhibited by each parasite strain. However, without targeted molecular studies, the role of hexose transporters in glucose acquisition by intracellular *T*. *cruzi* amastigotes versus possible alternative mechanisms such as fluid-phase endocytosis through the amastigote cytostome [53] remains unresolved.

Consistent with the potential for mammalian cells to sense fuel imbalances that may be incurred with the acquisition of glucose and other carbons by resident intracellular parasites and to mount a compensatory response [37, 41], we find that parasitized cells have more mitochondria and increased basal respiration. Selective inhibition of *T*. *cruzi* amastigote respiration with small molecule inhibitors of parasite cytochrome *bc*_*1*_, ELQ300 [43] and GNF7686 [47], enabled us to distinguish between parasite and host respiration and demonstrate elevated respiratory rates of mammalian cells during infection. However, unlike the impact of glucose restriction and/or 2-DG on amastigote proliferation, reduced host cell respiration, as seen in the mitochondrial complex III-deficient lines, did not impact *T*. *cruzi* replication. Infection outcomes are also anticipated to be host cell type–and perhaps parasite strain–dependent, as increased mitochondrial respiration in *T*. *cruzi*-infected macrophages was previously shown to be associated with increased nitric oxide production and parasite clearance [54], while our results show no association in non-phagocytic cells. Additional studies are needed to better understand the complex interplay between *T*. *cruzi* and host metabolism at the cellular, organ and organismal levels. How metabolic changes incurred at the cellular level impact regional and global metabolism in infected mammalian hosts [26, 27, 30–32, 55] and vice versa and how these changes impact the pathophysiology of disease are critical questions for future investigation.

In summary, we demonstrate that *T*. *cruzi* infection modulates host cell metabolism, stimulating glucose uptake into infected monolayers, which can be scavenged directly by intracellular amastigotes for utilization in energy generating and biosynthetic processes. Thus, in addition to amino acids and fatty acids predicted to constitute the main intracellular source of carbon for *T*. *cruzi* amastigotes [13, 18], we propose that glucose offers additional flexibility with respect to fuel utilization by these intracellular parasites. While the exact degree of *T*. *cruzi* amastigote metabolic plasticity has yet to be determined, a greater number of nutrient options is predicted to enhance the chances of parasite survival in different host tissues and under varying environmental conditions, including pharmacological inhibition of specific metabolic pathways.

## Materials and methods

### Parasite and mammalian cell culture

Mammalian cell lines: mouse skeletal muscle myoblast (C2C12; ATCC #CRL-1772), African green monkey kidney epithelial (LLcMK2; ATCC #CCL-7) and human dermal fibroblasts (NHDF; ATCC #CRL-2522 and NHDF-Neo; Lonza, #CC-2509) were propagated in Dulbecco’s Modified Eagle Medium (DMEM; Hyclone) supplemented with 1 mM pyruvate, 25 mM glucose, 2 mM glutamine, 100 U/ml penicillin, 10 μg/ml streptomycin and 10% fetal bovine serum (FBS) (D-10) at 37°C and 5% CO_2_. Human patient dermal fibroblast lines were purchased from the Cell line and DNA Bank of Genetic Movement Disorders and Mitochondrial Diseases (GMD-MDbank): Complex III mutant fibroblast harbor a mutation in subunit *BCS1L* of ETC complex III [45] (CIII mutant; GMD-MDbank #F-MT2614), Normal 1 fibroblast (GMD-MDbank #F-CR2631; Normal 2 fibroblast (GMD-MDbank #F-CR2571) were propagated in D-10 medium containing 50 μg/mL uridine (Sigma-Aldrich) at 37°C and 5% CO_2_.

#### Parasites

*Trypanosoma cruzi* Tulahuén strain [5] (ATCC #PRA-330), CL Brener strain [56] and the closely related avirulent CL-14 strain [57], (generously provided by R. Gazzinelli) were maintained by weekly passage in LLcMK2 cells in DMEM supplemented with 1 mM pyruvate, 25 mM glucose, 2 mM glutamine, 100 U/ml penicillin, 10 μg/ml streptomycin and 2% FBS (D-2) at 37°C, 5% CO_2_ as previously described [12]. Motile extracellular *T*. *cruzi* trypomastigotes were collected from infected LLcMK2 supernatants, pelleted at 2,000 *g* for 10 min and allowed to swim up from the pellet for a minimum of 2 h at 37°C, 5% CO_2_ before collection and use for infections. For all experimental *T*. *cruzi* infections, mammalian cells were grown in D-10 for 24 h to achieve ~80% confluence prior to incubation with freshly isolated trypomastigotes in D-2, followed by multiple washes in PBS (Gibco) to remove remaining extracellular trypomastigotes and further incubation in D-2 to allow intracellular development and growth of *T*. *cruzi* amastigotes for the indicated time periods. Tulahuén strain parasites were used unless otherwise indicated. *T*. *cruzi* epimastigotes were maintained axenically in liver infusion tryptose (LIT; 1% liver infusion broth, 68.4 mM sodium chloride, 5.4 mM potassium chloride, 5.5 mM glucose, 0.5% bacto-tryptose, 56.4 mM sodium phosphate dibasic, 0.002% hemin, 10% FBS, and 100 U/mL penicillin, and 100 μg/mL streptomycin) at 27°C.

### Construction of transgenic mammalian and parasite lines

Mammalian cell lines expressing mCherry targeted to the mitochondrial matrix were generated by retroviral transduction of NHDF and C2C12 with a construct containing the sequence encoding the first 25 amino acids of the mouse Cox8a protein fused to mCherry [58, 59]. Briefly, 5 x 10^5^ Phoenix-AMPHO packaging cells (ATCC #CRL-3213) were plated in a 100 mm tissue culture dish and transfected the following day with 10 μg of the plasmid pLNCX2 containing the chimeric sequence (kindly provided by C-H Lee, HSPH) using TransIT-LT1 (Mirus Bio) per manufacturer’s protocol. Virion-containing medium obtained from Phoenix cell cultures 2 days post-transfection was passed through a 0.45 μm filter, and stored at -80°C. Mammalian cells were seeded 1.5 x 10^5^ per well in a 6 well plate, and viron-containing medium with 4 μg/mL polybrene was added the following day. Transgenic fibroblasts were selected with 400 μg/mL G418 (Sigma-Aldrich) starting two days post-transduction and confirmed by microscopy and flow cytometry after 2 weeks.

*Trypanosoma cruzi* Tulahuén strain parasites (from M. Perrin, Tufts University) were transfected with pROCK-GFP-NEO for constitutive expression of GFP from the tubulin locus [60]. Axenically-grown *T*. *cruzi* epimastigotes were transfected as described [61] with minor alterations. Briefly, 10 μg of linearized plasmid was transfected into 4 x 10^7^ epimastigotes in 100 μL of Tb BSF-buffer using the U-33 program of an Amaxa Nucleofector II (Lonza). Cells were subsequently transferred to 5 mL LIT and incubated at 27°C overnight, then cloned in 96-well plates. After 3 weeks, clones were screened by flow cytometry, and GFP expressing parasites were confirmed by microscopy and PCR amplification of a 655 bp GFP sequence (forward: 5’-TTCACTGGAGTTGTCC-3’; reverse: 5’-AGTTCATCCATGCCAT-3’) and 772 bp Neo^r^ sequence (forward: 5’-ATGGGATCGGCCATT-3’; reverse: 5’-TCAGAAGAACTCGTCAAG-3’) using Taq DNA polymerase (GenScript) per manufacturer protocol. To generate trypomastigotes, 1 mL of stationary phase epimastigotes was added to a T75 flask of confluent LLcMK_2_ cells. The medium was changed to fresh D2 every day, and after 1 week newly emerging trypomastigotes were collected and used to start new mammalian stage cultures. Constitutive GFP expression in the parasite population was confirmed by routine fluorescence microscopy (Nikon TE-300).

### Isolation of intracellular *T*. *cruzi* amastigotes

NHDF were plated at 1.5 x 10^6^ in T75 flasks and infected with multiplicity of infection (MOI) of 10 for 18–24 h. At 48 hpi, monolayers were scraped and amastigotes were released from disrupted host cells by syringe passage (28^1/2^G needle; BD) into the indicated ice-cold buffer. Amastigotes were purified from debris by passage through a PD-10 desalting column (GE Healthcare Life Sciences), and fractions containing clean amastigotes were centrifuged at 4000 *g* for 10 minutes at 4°C to pellet amastigotes, which were resuspended in warm (37°C) buffer as needed for indicated applications.

### Genomic DNA PCR

NHDF were plated and infected as for mammalian glucose uptake assays (described below). At 48 hpi genomic DNA was isolated using the DNeasy kit (Qiagen) and eluted in water. 1 μL of sample was combined with 10 μL of iTaq Universal SYBR Green Supermix (Bio-Rad) and 5 μM of each human TNF primer (forward: 5’-TAAGATCCCTCGGACCCAGT-3’; reverse: 5’-GCAACAGCCGGAAATCTCAC-3’) in a 20 μL reaction, run as above, and analyzed using the default Standard Curve (absolute quantitation) settings of a StepOnePlus.

### Glucose transport assays

All transport assays were performed using 1,2-^3^H(N)-2-deoxyglucose, ([^3^H]-2-DG; PerkinElmer).

#### Mammalian cells

NHDF were plated at 1 x 10^5^ per well in 12 well plates and incubated with T. cruzi trypomastigotes at the indicated MOI or MOI of 40 for 1 hour. At 48 hpi monolayers were washed twice with Krebs-Ringer bicarbonate HEPES buffer (KRBH; 120 mM sodium chloride, 4 mM potassium phosphate monobasic, 1 mM magnesium sulfate, 0.75 mM calcium chloride, 30 mM HEPES, and 10 mM sodium bicarbonate) and incubated in 400 μL fresh KRBH at 37°C for 10 minutes. Transport assays were started by the addition of 0.5 μCi [^3^H]-2-DG and a final concentration of 55.5 μM unlabeled 2-DG and incubated at 37°C for 20 minutes. The reaction was stopped by addition of 35 μM cytochalasin B (MP Biomedicals) and monolayers were washed 5 times to remove residual 2-DG. All cells were lysed in 500 μL of 0.1 M sodium hydroxide at room temperature for 10 minutes, and 400 μL was added to 5 mL of Ecolite(+) (MP Biomedicals) for scintillation counting in a Beckman LS6500 scintillation counter. Background counts were removed by subtracting the measurements from samples where [^3^H]-2-DG and cytochalasin B were added simultaneously before immediate washing.

#### *In situ* amastigotes

NHDF were seeded at 1.5 x 10^6^ in T75 flasks and infected with MOI of 10 for 18 h. The assay was conducted as above using 10 μCi of [^3^H]-2-DG and 83.3 μM unlabeled 2-DG to start transport. To inhibit mammalian cell glucose transport, cytochalasin B was added with 2-DG to a final concentration of 15 μM. The reaction was stopped as above and monolayers were washed 5 times rapidly to remove exogenous [^3^H]-2-DG. Amastigotes were isolated from host cell monolayers in Krebs-Henseleit Buffer (KHB; 111 mM sodium chloride, 4.7 mM potassium chloride, 1.25 mM calcium chloride, 2 mM magnesium sulfate, and 1.2 mM sodium phosphate dibasic) then lysed for scintillation counts and protein normalization. To verify that the intracellular parasites had taken up [^3^H]-2-DG label from host cell, amastigotes were isolated following radiolabeling of infected monolayers as above, split equally into two tubes and incubated with vehicle alone (1% DMSO) or 0.05 mg/mL alamethicin (VWR) in 500 μL for 15 minutes at room temperature while shaking to permeabilize the parasite plasma membrane. An additional 500 μL of buffer was added, and amastigotes were pelleted at 4000 *g* for 10 minutes at 4°C and lysed in sodium hydroxide for scintillation counts.

#### Isolated parasites

For transport assays in isolated parasites, trypomastigotes were washed twice and assayed in KHB and amastigotes were isolated and assayed in KHB or cytobuffer (10 mM sodium chloride, 140 mM potassium chloride, 2 mM magnesium chloride, 2 μM calcium chloride, and 10 mM HEPES at pH 7.4) meant to mimic intracellular ion concentrations [56]. As determined subsequently, amastigote glucose transport capacity was similar in cytobuffer and KHB. Parasites were aliquoted at 5 x 10^6^ in 100 μL and 100 μL of loading solution (0.5 μCi [^3^H]-2-DG in 2x the indicated final 2-DG concentration) was added. After 1 minute, 800 μL of ice-cold buffer supplemented with 25 mM glucose was added and the tube was placed on ice to stop transport. Parasites were washed twice by centrifugation at 4000 *g* for 10 minutes at 4°C and resuspended in ice-cold buffer with 25 mM glucose, then lysed in sodium hydroxide following centrifugation. 400 μL were used for scintillation counts and 10 μL were used for protein normalization by comparison to an IgG standard curve using the Peirce 660nm Protein Assay Reagent (Thermo Fisher Scientific) per manufacturer protocol. Background counts were removed by subtracting the measurements of samples where transport was prevented by adding ice-cold buffer with 25 mM glucose and [^3^H]-2-DG simultaneously. To calculate the number of moles of 2-DG/counts per minute (cpm), the total concentration of 2-DG in each loading solution was multiplied by the volume and divided by the cpm of 100 μL of each loading solution. V_0_ was calculated by multiplying that number by the measurement of cpm/mg protein for each sample.

### Lactate determination

The wells of an XF^e^24 cell culture microplate (Agilent Technologies) were coated with 0.1% gelatin and incubated at 37°C for 1 hour before gelatin was aspirated and NHDF were plated at 1.5 x 10^4^ in 250 μL. Cells were infected with MOI of 50 for 1 hour then placed in 250 μL D-2. At 48 hpi media was changed to Seahorse XF base medium without phenol red (DMEM-based medium; Agilent Technologies), supplemented with 2 mM glucose and 10 mM glutamine, by rinsing cells twice with 1 mL medium, then replacing it with 500 μL medium. After 1 hour of incubation at 37°C, the supernatant was collected and 2 μL of sample was assayed by lactate assay kit I (Biovision) per manufacturer protocol.

### Seahorse bioenergetics analysis

#### Mammalian cells

NHDF were plated and infected as in the lactate assay. Seahorse XF Base Medium was supplemented with 1 mM sodium pyruvate, 2 mM glutamine, and 10 mM glucose (XBMS; Agilent Technologies). Cells were rinsed with XBMS as above, then incubated in 400 μL at 37°C without CO_2_ for 45 minutes. ELQ300 (generously provided by M. Riscoe, OHSU), GNF7686 (Lab Network), or equivalent vehicle was added directly to cell monolayers to a final concentration of 1 μM in 450 μL and the plate was incubated at 37°C without CO_2_ for 15 minutes prior to initiation of assay. Compounds from the Seahorse XF Cell Mito Stress Test (Agilent Technologies) were resuspended in XMBS per manufacturer protocol, then diluted to 10x final concentrations and loaded into the sensor cartridge ports for injection. Final concentrations were 1 μM oligomycin (O), which inhibits ATP synthase, 2.5 μM FCCP (F), which uncouples ETC activity from ATP synthesis, and 1 μM each of rotenone and antimycin A (R/A), which inhibit ETC complex I and III respectively. The Seahorse XF^e^24 was programed to mix for 3 minutes, wait 2 minutes then obtain measurements for 3 minutes. Basal respiration was calculated as the difference between OCR at baseline and that after R/A injection. Spare respiratory capacity (SRC) was calculated as the difference between OCR after FCCP injection and that at baseline. Protein concentrations were calculated where indicated by lysing monolayers in 0.1 M sodium hydroxide and comparing protein abundance to an IgG standard curve by Bradford assay (Bio-Rad).

#### Isolated amastigotes

*T*. *cruzi* amastigotes were purified from infected host cell monolayers as above, then adhered to microplates with Cell-Tak (Corning) as described [30]. For the ELQ300 titration experiment (S1B Fig), amastigotes were isolated and assayed in XBMS and pretreated with the indicated amount of ELQ300. Oligomycin, FCCP, and rotenone and antimycin A were injected sequentially, and measurements were taken as previously described [17]. For substrate response assays (Fig 3A and 3B), amastigotes were isolated and assayed in KHB. During the assay, 2.5 mM glucose or glutamine, 100 mM 2-DG, and 1 μM each of rotenone and antimycin A was injected at the times indicated.

### Mitochondrial content by flow cytometry

NHDF or C2C12 stably expressing mCherry in the mitochondria were plated at 1.5 x 10^5^ per well and infected with MOI of 20–40 GFP-expressing parasites for 18 h or treated with the indicated concentration of valproic acid starting from the time of infection. All centrifugation steps were carried out at room temperature at 350 *g* for 5 minutes. At 48 or 66 hpi, infected cells were trypsinized and centrifuged, then resuspended in 4% paraformaldehyde in PBS to fix on ice for 20 minutes. Cells were recentrifuged and the pellet was permeabilized in PBS + 0.1% Triton X-100 (PBST) with 0.5 μg/mL DAPI. Samples were run on a MACSQuant VYB and data was analyzed using FlowJo 7.6. Cells were discriminated based on size and DAPI staining. Infected cells were identified based on GFP expression (S2D Fig), and mCherry expression was examined for all populations. At least 10,000 events were collected per condition.

Flow cytometric analysis of an endogenous mitochondrial marker, was carried out using antibodies to ATP5B (Abcam #3D5) to stain infected and uninfected NHDF (without mCherry) as above. Following permeabilization for 20 min at room temperature, cells were centrifuged and resuspended in blocking solution (1% bovine serum albumin in PBS) and incubated for 30 minutes at room temperature. After centrifugation, cells were incubated for 30 minutes at room temperature with mouse anti-ATP5B (Abcam #3D5) at 1:2,000 in blocking solution then washed twice in PBST and incubated in 1:2,500 AlexaFluor 594 Goat anti-Mouse IgG (Thermo Fisher Scientific) in blocking solution for 30 minutes, washed as with primary antibody and resuspended in PBST with 0.5 μg/mL DAPI for flow cytometry analysis as above.

### Western blot

NHDF were seeded at 1.5 x 10^6^ in T75 flasks and infected with a MOI of 10 for 2 hours. At 48 hpi, cells were scraped, pelleted by centrifugation and resuspended in Laemmli SDS-PAGE sample buffer at 2 x 10^4^ cells/μL. Cell lysates were treated with 0.5 μL benzonase to digest DNA, incubated on ice 30 minutes, then at 95°C for 10 minutes before clarification by centrifugation at 15,000 *g* for 10 minutes. Due to the presence of parasites in infected cells compared to mock-infected cells, we could not load gels based on protein quantification. Instead, 2 x 10^5^ cells (~30 μg of uninfected cells) were loaded in wells of a 4–15% Mini-Protean TGX Precast Gel (Bio-Rad) and the Western gel and wet transfer to a PVDF membrane was done per manufacturer protocol. All incubation and wash steps were done while shaking. The membrane was blocked in 1:1 SeaBlock:PBS (Thermo Fisher Scientific) for 1 hour at room temperature, incubated overnight at 4°C with the corresponding antibodies in 1:1 SeaBlock:PBS-0.1% Tween-20, then washed three times in PBS-0.1% Tween-20 prior to incubation with secondary antibodies in 1:1 SeaBlock:PBS-0.1% Tween-20 for 30 min at room temperature. The membrane was washed 3 times briefly in PBS-0.1% Tween-20 and 3 times in PBS for 10 minutes before imaging using an Odyssey Imaging System (Li-cor). The intensity of the signal for each antibody was assessed by Image Studio software (version 5.2). Lack of cross-reactivity of the antibodies with amastigote proteins was verified using amastigotes lysate following amastigote purification. Vimentin was used as a loading control. Primary antibodies: TOMM20 (F-10, Santa Cruz; 1:1,000), ATP5B (3D5, Abcam; 1:1,000), vimentin (5741, Cell Signaling; 1:2,000). Secondary antibodies: Dylight 800 anti-rabbit (Invitrogen, 1:10,000), Dylight 800 anti-mouse (Invitrogen, 1:5,000), Dylight 700 anti-rabbit (Invitrogen, 1:20,000).

### *T*. *cruzi* amastigote proliferation assay

To determine the number of divisions that an intracellular *T*. *cruzi* amastigote has undergone in a defined period of time, a modified flow cytometry protocol, based on [34] was performed. Briefly, NHDF were plated at 1.5 x 10^5^ per well in 6 well plates and infected with MOI of 15 for 2 hours using CFSE-stained trypomastigotes. For staining, 5 x 10^6^ trypomastigotes/mL were stained with 1 μM CFSE (Thermo Fisher Scientific) in PBS by incubating at 37°C for 15 minutes. Extra dye was quenched by addition of D-10, and trypomastigotes were pelleted by centrifugation at 2100 *g* for 10 minutes and incubated in fresh D-10 at 37°C for 30 minutes before infection. At 18 (pre-replication) and 48 (replicative phase) hpi, infected monolayers were trypsinized, washed once in PBS, and cells were lysed to release amastigotes by passing the supernatant 10 times through a 28^1/2^G needle. Lysate was fixed by adding paraformaldehyde (Electron Microscopy Sciences) to a final concentration of 1% and incubating 20 minutes on ice. Samples were centrifuged at 300 *g* for 5 minutes to pellet away host debris, and the supernatant centrifuged at 4000 *g* for 10 minutes to pellet amastigotes. Pellets were resuspended in PBS with 0.1% Triton X-100 and 0.01 μg/mL DAPI for analysis by flow cytometry. Amastigotes were run on a MACSQuant VYB (Miltenyi Biotec) or LSRII (BD Biosciences) and at least 10,000 events were collected per condition. Data was analyzed using FlowJo 7.6, and amastigotes were discriminated based on size and DAPI staining. Proliferation was modeled using FlowJo 7.6, and CFSE intensity at 18 hpi was set as peak 0 for all samples.

### Multiplexed *T*. *cruzi* infection assay

Host cell and intracellular *T*. *cruzi* amastigote numbers were assessed as described with minor modifications [34]. Briefly, NHDF were plated at 1.5 x 10^3^ per well in 384 well plates and infected with MOI 1.25 for 2 hours before incubation in phenol-free media at the indicated glucose concentration. At 18 hpi, the indicated concentration of 2-DG was added, and at 66 hpi media was removed and host cell number and parasite number were assessed using 10 μL of CellTiter-Fluor (Promega) and 10 μL of Beta-Glo (Promega) per well, respectively.

### Metabolite profiling

Isolated amastigotes were resuspended in cytobuffer at 2 x 10^7^ parasites/mL and incubated for 3 hours at 37°C with 6 mM U-^13^C-glucose (Cambridge Isotope Laboratories) or unlabeled glucose (Sigma Aldrich). For metabolite extraction, amastigotes were rapidly cooled to 4°C in a dry-ice ethanol bath with gentle agitation as described [62], then centrifuged at 3200 *g* for 10 minutes at 4°C and resuspended in 80% (v/v) methanol:water to extract metabolites from the pellet as described [63]. Samples were run in technical triplicate and metabolites detected by the Beth Israel Deaconess Medical Center Mass Spectrometry Facility as described [64–66]. The percent of label incorporation was calculated for each replicate as the peak area of all ^13^C-labeled variants of a metabolite divided by the sum of both the labeled- and unlabeled-metabolite peak areas. Background was subtracted by averaging the percent label incorporation of unlabeled-replicates and subtracting that value from each labeled replicate.

### ATP assay

Amastigotes were isolated in KHB and incubated at 4 x 10^5^ amastigotes/mL in 5 mM glucose, 5mM glutamine, or 1 mM pyruvate as indicated at 37°C. Total ATP content was measured in isolated *T*. *cruzi* amastigotes under each condition at 0 hr and 24 hr using the ATPlite assay (PerkinElmer) following the manufacturer’s protocol.

### Reverse transcription and qPCR

RNA was extracted from either trypomastigotes or purified amastigotes (48 hpi) using the RNeasy purification kit (Qiagen). Using 500ng RNA, cDNA was generated through the iScript cDNA Synthesis Kit (Bio-Rad). Primer sets used for amplification of TcHT were TcHT-F: 5’-TGATGTACCATGTGTCCTCGGCAACG-3’ and TcHT-R 5’-ATGGCACTGCGCTGGACCCGA-3’ [15] or TcHT-F2: 5’-TCCTTCGTGCTCCTGACGAATT-3’ and TcHT-R2: 5’-AAAAGATGAACGCGACTGCCTG-3’. The primer set for amplification of a parasite housekeeping, ribosomal RNA large subunit gamma M1 was Ribo-F: 5’ -TGTGGAAATGCGAAACAC-3’ and Ribo-R: 5’-CCCAGGTTTTTGCTTTAATG-3’ [12]. Thermal cycling proceeded at 95°C for 10 minutes followed by forty cycles of 95°C 15 seconds and 60°C 1 minute using a StepOnePlus Real-Time PCR System (Applied Biosystems). Relative TcHT abundance was measured using SYBR green iTaq Universal Mix (Bio-Rad) and calculated using the ribosomal control and ΔΔCt method.

### Microscopy

NHDF were seeded at 5 x 10^4^ per well in 24 well plates on 12 mm round German glass coverslips (Electron Microscopy Services) and infected with MOI of 10 for 1 hour on glass coverslips. At 48 and 66 hpi, coverslips were fixed in 4% paraformaldehyde in PBS overnight at 4°C, then stained with 2.5 μg/mL DAPI and mounted on slides in Mowiol mounting medium. Slides were imaged using a Nikon TE300 and the number of amastigotes per cell was counted for 100 cells per condition.

### Statistical analysis

Figures presented show mean values with standard deviation of biological replicates or medians (non-parametric data). Independent experiments were compared where indicated. Comparison of more than two groups was performed using a One-way ANOVA for single factor experiments and Two-way ANOVA for comparisons with two independent variables. The non-parametric Kruskal-Wallis test was used for comparisons of more than two groups that did not have normal distributions. If significant, post hoc tests were used (p values indicated) to compare specific groups and correct for multiple comparisons between groups. Statistical analysis was performed using Prism 7 (GraphPad).

## Supporting information

S1 Fig Specific targeting of *T*. *cruzi* amastigote mitochondrial respiration *in situ* with ELQ300 and GNF7686. **(A)** Quantitative PCR analysis of genomic DNA isolated from NHDF monolayers infected with different multiplicity of infection indicate no difference in host cell abundance due to infection at 48 hpi. Mean ± SD shown for 3 biological replicates. **(B)** Dose-dependent inhibition of mitochondrial respiration (OCR) from isolated *T*. *cruzi* amastigotes with ELQ300. Maximal effect of >90% inhibition achieved with 1 μM ELQ300. Mean ± SD shown for 3 biological replicates. One-way ANOVA with Dunnett’s multiple comparisons test was applied for individual comparisons to vehicle control (**p< 0.01, ***p< 0.001). **(C)** ELQ300 does not inhibit host cell (NHDF) mitochondrial respiration at 10 μM. Mean ± SD shown for 3 biological replicates. Student’s t-test was applied. **(D)** Complex III-deficient human dermal fibroblasts (CIII mutant) display reduced basal OCR and a limited response to FCCP as compared to two independent normal fibroblast control lines, as determined using the Mito Stress Test which involves sequential injection of oligomycin (O), FCCP (F) and rotenone/antimycin A (R/A) (as detailed in Methods). **(E-F)** 1 μM ELQ300 pre-treatment (30 minutes) of uninfected and infected fibroblasts (48 hpi) inhibited parasite-specific respiration in CIII mutant fibroblasts reducing OCR to the level of uninfected cells. Parallel experiments conducted in two normal human fibroblast lines (Normal 1, Normal 2) revealed residual OCR following inhibition of parasite respiration that is attributable to the host cell. Mean ± SD shown for 3 biological replicates. Two-way ANOVA with Tukey’s multiple comparisons test was applied for individual comparisons (*p< 0.05, **p< 0.01, ***p< 0.001, ****p< 0.0001). **(G)** ATP levels measured in freshly isolated *T*. *cruzi* amastigotes in KHB pH 7.2 with 2 mM glutamine following incubation with antimycin A (AA), ELQ300, or GNF7686 for 30 minutes at the indicated concentrations. Data is represented relative to vehicle control. Mean ± SD shown for 3 biological replicates. **(H)** Oxygen consumption rates (OCR) measured in uninfected and infected NHDF monolayers (48hpi) following a 30 minute pretreatment with 1 μM ELQ300, 1 μM GNF7686, or vehicle control. Basal OCR measurements were established before sequential injection of oligomycin (O) and rotenone and antimycin A (R/A) to inhibit ATP-linked respiration and total mitochondrial respiration respectively. Mean ± SD shown for 3 biological replicates. **(I)** GNF7686 and ELQ300 have a similar capacity to inhibit parasite respiration in infected monolayers. *T*. *cruzi*-infected NHDF monolayers were treated with 1 μM ELQ300 or 1 μM GNF7686 to selectively remove amastigote respiration from the total OCR signal and reveal increased host respiration during *T*. *cruzi* infection. Mean ± SD shown for 3 biological replicates. Two-way ANOVA with Tukey’s multiple comparisons test was applied for individual comparisons (***p< 0.001, ****p< 0.0001).
(TIF)

S2 Fig Increased mitochondrial content in *T*. *cruzi*-infected host cells. **(A)** Western blots of endogenous mitochondrial protein expression, ATP5B and TOMM20, relative to vimentin in total lysates prepared from uninfected (UN), infected (INF), or valproic acid (VA) treated NHDF monolayers at 48 hpi. Valproic acid treatment was included as a positive control for mitochondrial biogenesis [67]. (**B**) Graphs show the mean ± SD of the relative intensity of ATP5B or TOMM20 versus vimentin in uninfected controls compared to parasite-infected or VA-treated cells from 4 independent experiments. One-way ANOVA with Dunnett’s multiple comparisons test was applied (*p< 0.05. **p< 0.01). **(C)** Dose-response of NHDF-mito-mCherry to valproic acid treatment for 48 h demonstrates utility of mito-mCherry for measuring changes in mitochondrial content by flow cytometry. **(D)** NHDF-mito-mCherry monolayers were infected with *T*. *cruzi* expressing GFP and harvested at 48 or 66 hpi. Cells were gated on side scatter (SSC) and GFP fluorescence, allowing for identification of parasitized cells from infected samples. **(E)** Flow cytometric detection of mCherry fluorescence in uninfected NHDF-mito-mCherry monolayers and in the parasitized (GFP+) and parasite-free (GFP-) subpopulations of an infected NHDF-mito-mCherry monolayer at 48 hpi and 66 hpi. Valproic acid (10 mM) treatment was included as a positive control for mitochondrial biogenesis.
(TIF)

S3 Fig Inhibition of intracellular *T*. *cruzi* proliferation by 2-deoxyglucose. **(A)** Relative host cell abundance at 66 hpi (CellTiter-Fluor, CTF) for NHDF cultured with the indicated concentration of 2-DG starting at 18 hpi. Mean ± SD of 4 biological replicates shown with nonlinear fit using log(inhibitor) vs. response with variable slope. **(B)** Microscopic counts of the number of intracellular *T*. *cruzi* amastigotes in infected NHDF cultured in varying concentrations of glucose ± 2 mM 2-DG beginning at 18 hpi. At 66 hpi, infected cells were fixed and DAPI-stained for microscopy. The median number of intracellular amastigotes per infected host cell in each condition is indicated by horizontal black bars. Significant differences between conditions were determined using Kruskal-Wallis with Dunn’s multiple comparison test (***p< 0.001, ****p< 0.0001).
(TIF)

S4 Fig *T*. *cruzi* amastigotes access glucose *in situ*. **(A)** Isolated amastigotes were treated with alamethicin and processed for flow cytometric determination of permeabilization using propidium idodide (PI) exclusion as previously described [17]. Permeabilization with 4% PFA was used as a positive control. **(B-D)** Incorporation of exogenous [^3^H]-2-DG by intracellular *T*. *cruzi* amastigotes *in situ* indicate that **(B)** Tulahuén amastigotes in C2C12, **(C)** CL Brener amastigotes in NHDF, and **(D)** CL-14 amastigotes in NHDF all access glucose *in situ*. *T*. *cruzi-*infected monolayers were incubated with 10 μCi [^3^H]-2-DG in the absence or presence of cytochalasin B (15 μM) for 20 minutes prior to isolation of intracellular amastigotes for scintillation counts, normalized to parasite protein (μg). Mean ± SD of 2 independent experiments. Student’s t-test was applied (*p< 0.05).
(TIF)

S5 Fig Quantification of *T*. *cruzi* hexose transporter abundance. Relative quantification of hexose transporter mRNA, relative to trypomastigotes using **(A)** previously published primers [15] (average Ct value of 32.0) and **(B)** an independent primer set (average Ct value of 21.6) for amplification. Amplification of ribosomal RNA was used as a loading control for ΔΔCt calculations. Mean ± SD shown for technical triplicates.
(TIF)
