# Supplementary figures and images for: Modulation of host central carbon metabolism and in situ glucose uptake by intracellular Trypanosoma cruzi amastigotes
Source: PLoS Pathog. 2017 Nov 27;13(11):e1006747. doi: 10.1371/journal.ppat.1006747 (PMC5720825; doi:10.1371/journal.ppat.1006747)

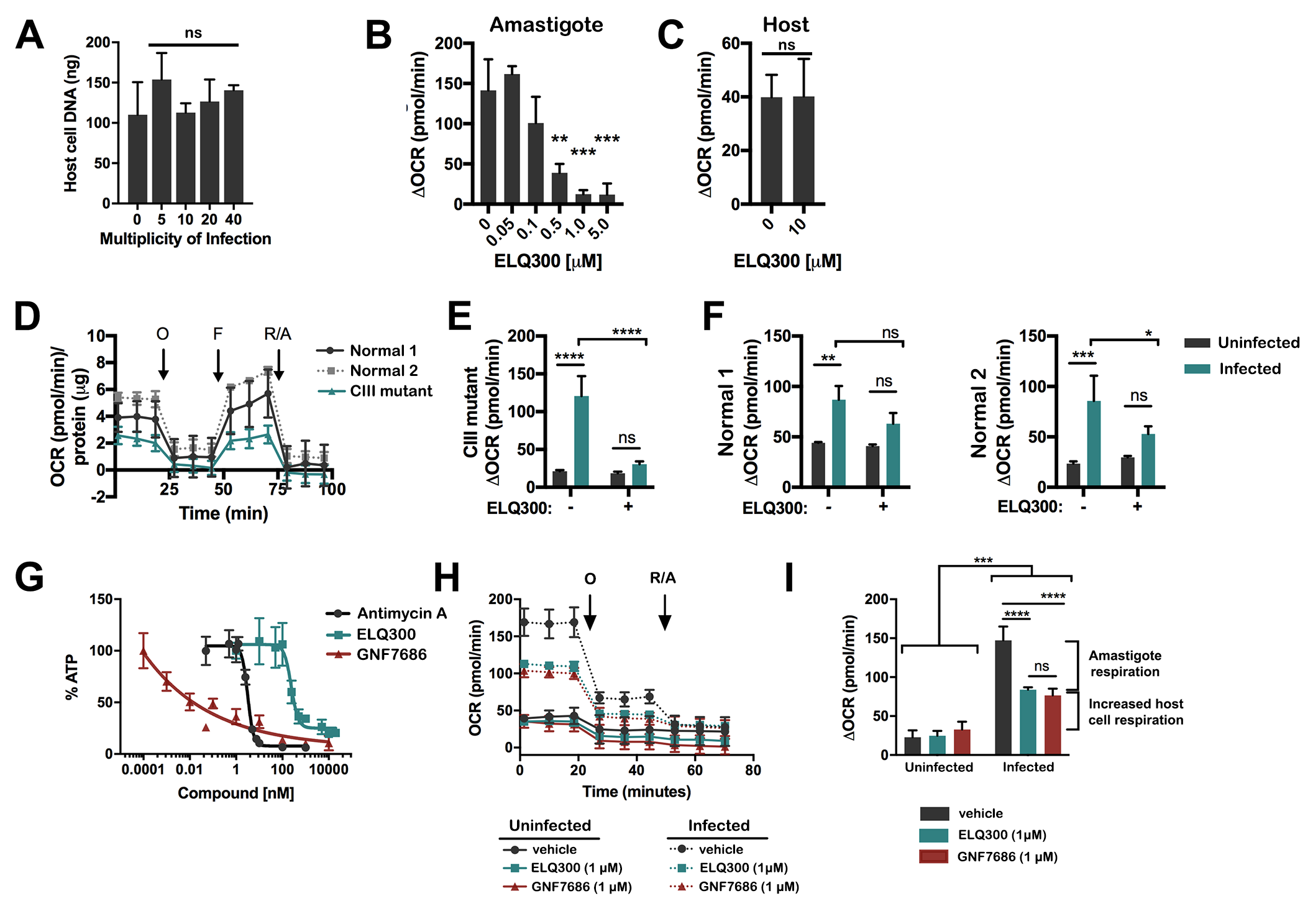

Supplement: S1 Fig — (A) Quantitative PCR analysis of genomic DNA isolated from NHDF monolayers infected with different multiplicity of infection indicate no difference in host cell abundance due to infection at 48 hpi. Mean ± SD shown for 3 biological replicates. (B) Dose-dependent inhibition of mitochondrial respiration (OCR) from isolated T. cruzi amastigotes with ELQ300. Maximal effect of >90% inhibition achieved with 1 μM ELQ300. Mean ± SD shown for 3 biological replicates. One-way ANOVA with Dunnett’s multiple comparisons test was applied for individual comparisons to vehicle control (**p< 0.01, ***p< 0.001). (C) ELQ300 does not inhibit host cell (NHDF) mitochondrial respiration at 10 μM. Mean ± SD shown for 3 biological replicates. Student’s t-test was applied. (D) Complex III-deficient human dermal fibroblasts (CIII mutant) display reduced basal OCR and a limited response to FCCP as compared to two independent normal fibroblast control lines, as determined using the Mito Stress Test which involves sequential injection of oligomycin (O), FCCP (F) and rotenone/antimycin A (R/A) (as detailed in Methods). (E-F) 1 μM ELQ300 pre-treatment (30 minutes) of uninfected and infected fibroblasts (48 hpi) inhibited parasite-specific respiration in CIII mutant fibroblasts reducing OCR to the level of uninfected cells. Parallel experiments conducted in two normal human fibroblast lines (Normal 1, Normal 2) revealed residual OCR following inhibition of parasite respiration that is attributable to the host cell. Mean ± SD shown for 3 biological replicates. Two-way ANOVA with Tukey’s multiple comparisons test was applied for individual comparisons (*p< 0.05, **p< 0.01, ***p< 0.001, ****p< 0.0001). (G) ATP levels measured in freshly isolated T. cruzi amastigotes in KHB pH 7.2 with 2 mM glutamine following incubation with antimycin A (AA), ELQ300, or GNF7686 for 30 minutes at the indicated concentrations. Data is represented relative to vehicle control. Mean ± SD shown for 3 biological replicates. [file ppat.1006747.s001.tif]

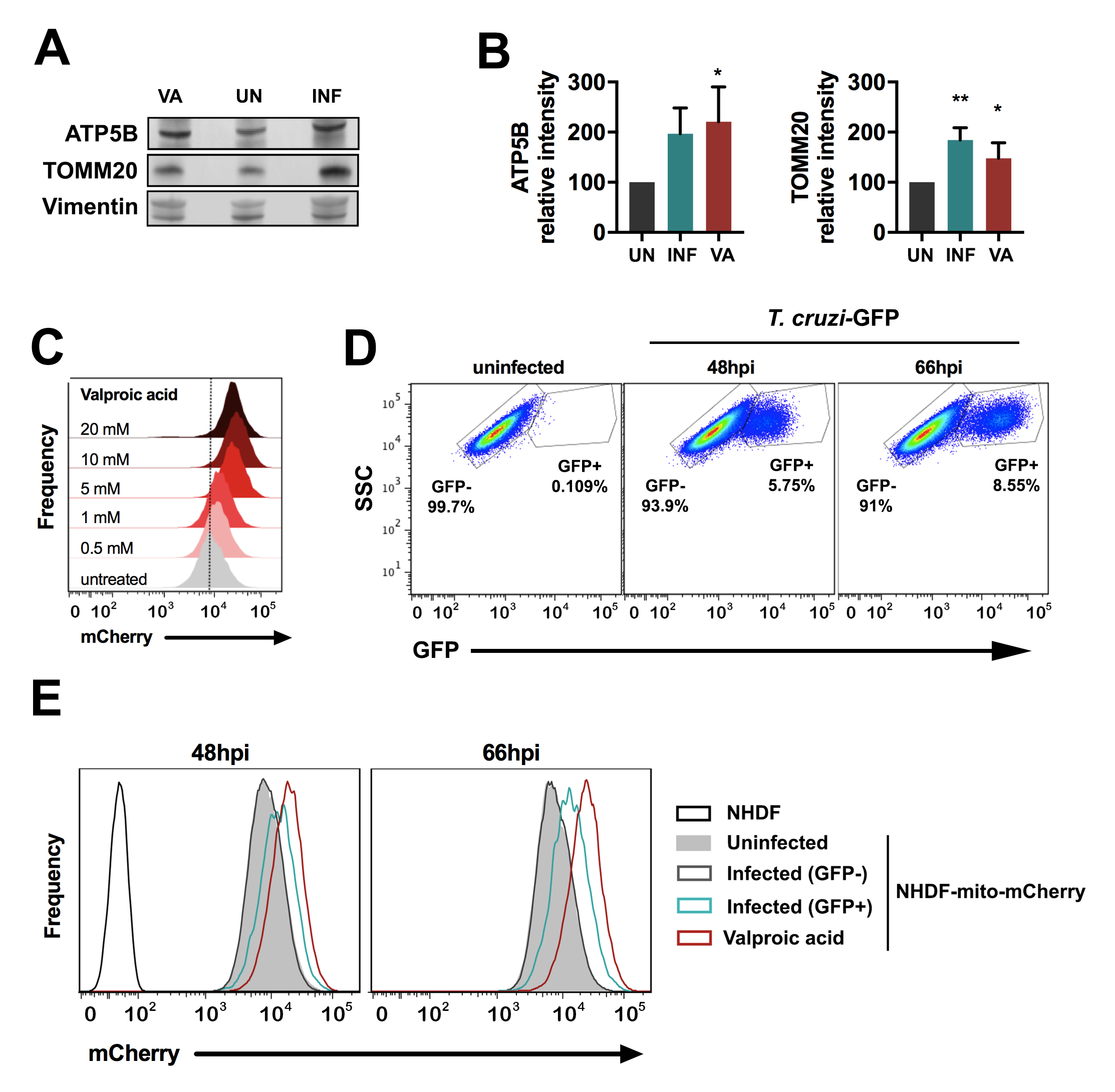

Supplement: S2 Fig — (A) Western blots of endogenous mitochondrial protein expression, ATP5B and TOMM20, relative to vimentin in total lysates prepared from uninfected (UN), infected (INF), or valproic acid (VA) treated NHDF monolayers at 48 hpi. Valproic acid treatment was included as a positive control for mitochondrial biogenesis [67]. (B) Graphs show the mean ± SD of the relative intensity of ATP5B or TOMM20 versus vimentin in uninfected controls compared to parasite-infected or VA-treated cells from 4 independent experiments. One-way ANOVA with Dunnett’s multiple comparisons test was applied (*p< 0.05. **p< 0.01). (C) Dose-response of NHDF-mito-mCherry to valproic acid treatment for 48 h demonstrates utility of mito-mCherry for measuring changes in mitochondrial content by flow cytometry. (D) NHDF-mito-mCherry monolayers were infected with T. cruzi expressing GFP and harvested at 48 or 66 hpi. Cells were gated on side scatter (SSC) and GFP fluorescence, allowing for identification of parasitized cells from infected samples. (E) Flow cytometric detection of mCherry fluorescence in uninfected NHDF-mito-mCherry monolayers and in the parasitized (GFP+) and parasite-free (GFP-) subpopulations of an infected NHDF-mito-mCherry monolayer at 48 hpi and 66 hpi. Valproic acid (10 mM) treatment was included as a positive control for mitochondrial biogenesis. (TIF) [file ppat.1006747.s002.tif]

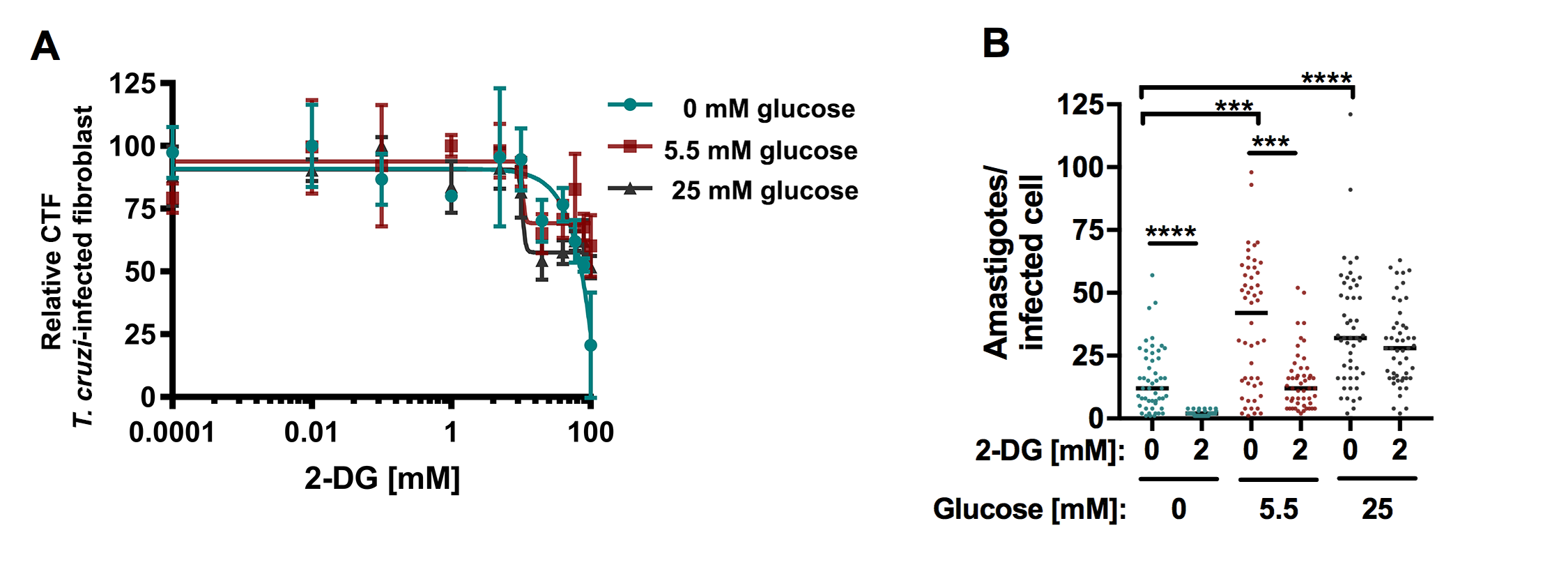

Supplement: S3 Fig — (A) Relative host cell abundance at 66 hpi (CellTiter-Fluor, CTF) for NHDF cultured with the indicated concentration of 2-DG starting at 18 hpi. Mean ± SD of 4 biological replicates shown with nonlinear fit using log(inhibitor) vs. response with variable slope. (B) Microscopic counts of the number of intracellular T. cruzi amastigotes in infected NHDF cultured in varying concentrations of glucose ± 2 mM 2-DG beginning at 18 hpi. At 66 hpi, infected cells were fixed and DAPI-stained for microscopy. The median number of intracellular amastigotes per infected host cell in each condition is indicated by horizontal black bars. Significant differences between conditions were determined using Kruskal-Wallis with Dunn’s multiple comparison test (***p< 0.001, ****p< 0.0001). (TIF) [file ppat.1006747.s003.tif]

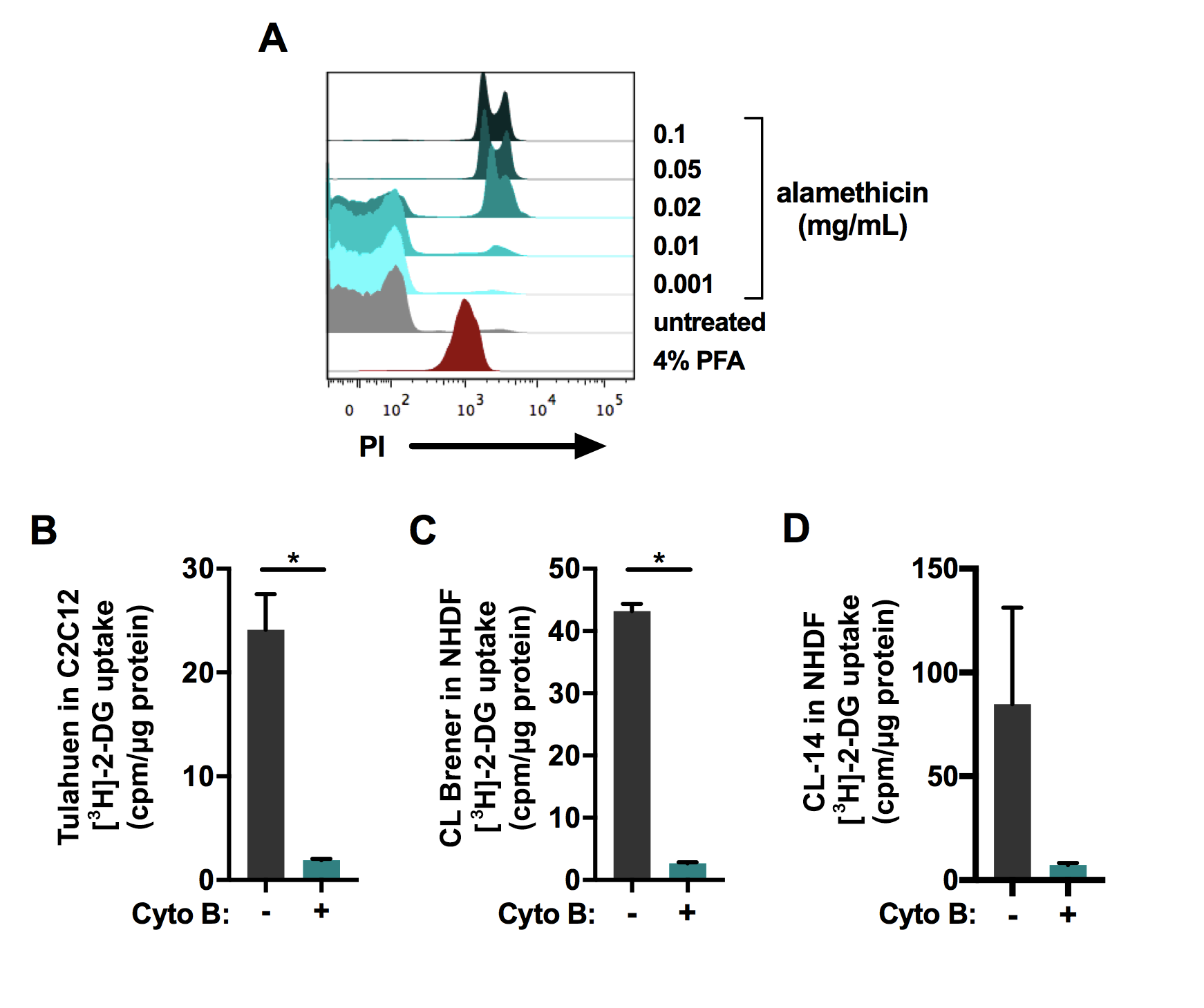

Supplement: S4 Fig — (A) Isolated amastigotes were treated with alamethicin and processed for flow cytometric determination of permeabilization using propidium idodide (PI) exclusion as previously described [17]. Permeabilization with 4% PFA was used as a positive control. (B-D) Incorporation of exogenous [3H]-2-DG by intracellular T. cruzi amastigotes in situ indicate that (B) Tulahuén amastigotes in C2C12, (C) CL Brener amastigotes in NHDF, and (D) CL-14 amastigotes in NHDF all access glucose in situ. T. cruzi-infected monolayers were incubated with 10 μCi [3H]-2-DG in the absence or presence of cytochalasin B (15 μM) for 20 minutes prior to isolation of intracellular amastigotes for scintillation counts, normalized to parasite protein (μg). Mean ± SD of 2 independent experiments. Student’s t-test was applied (*p< 0.05). (TIF) [file ppat.1006747.s004.tif]

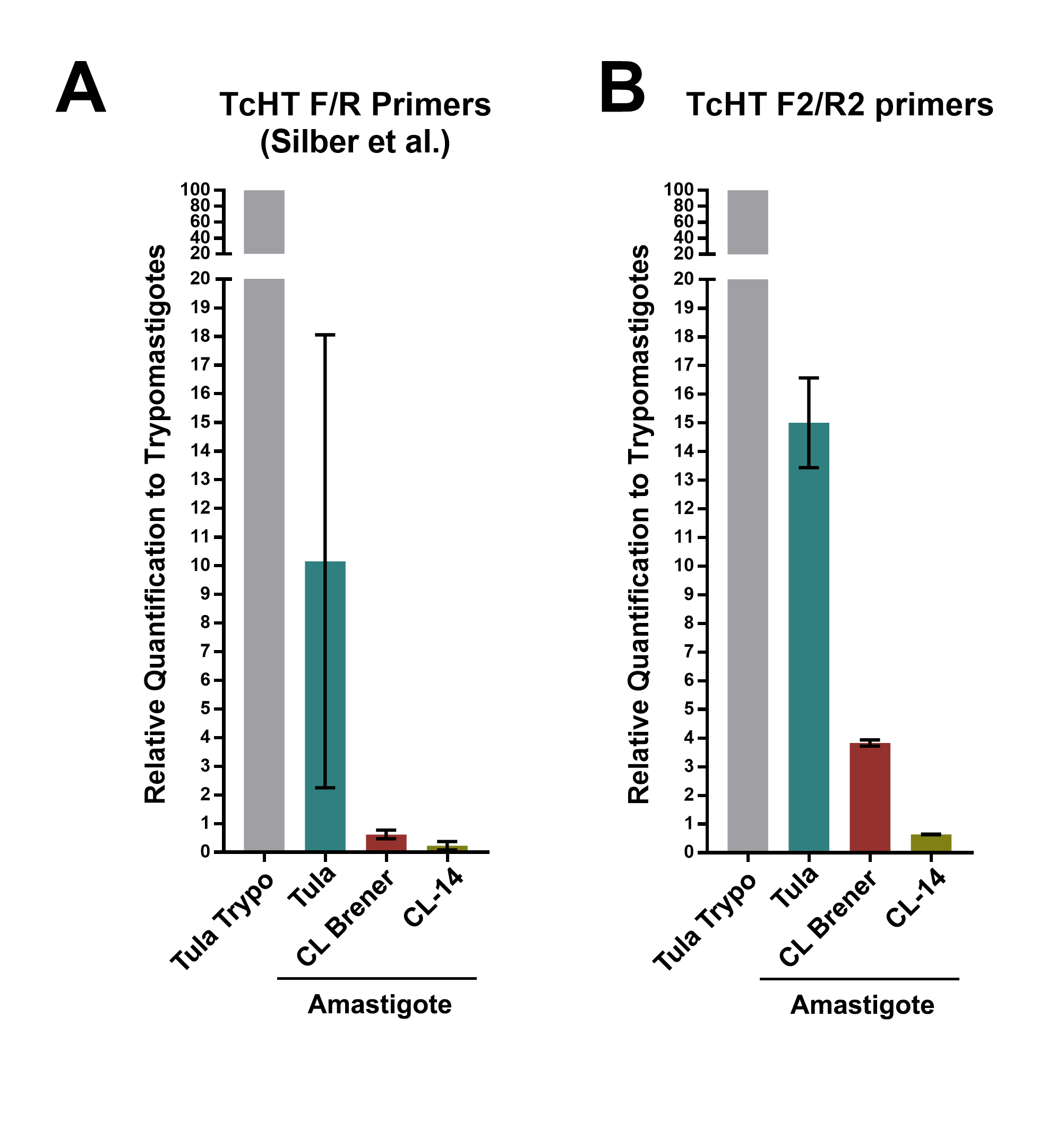

Supplement: S5 Fig — Relative quantification of hexose transporter mRNA, relative to trypomastigotes using (A) previously published primers [15] (average Ct value of 32.0) and (B) an independent primer set (average Ct value of 21.6) for amplification. Amplification of ribosomal RNA was used as a loading control for ΔΔCt calculations. Mean ± SD shown for technical triplicates. (TIF) [file ppat.1006747.s005.tif]
